# Sensorimotor integration and functional mobility in early-stage Parkinson’s disease: A cross-sectional study using clinically feasible assessments

**DOI:** 10.1371/journal.pone.0348668

**Published:** 2026-05-06

**Authors:** Mohammad A. ALMohiza, Ravi Shankar Reddy

**Affiliations:** 1 Department of Rehabilitation Sciences, College of Applied Medical Sciences, King Saud University, Riyadh, Saudi Arabia; 2 Program of Physical Therapy, Department of Medical Rehabilitation Sciences, College of Applied Medical Sciences, King Khalid University, Abha, Saudi Arabia; University of Illinois Urbana-Champaign, UNITED STATES OF AMERICA

## Abstract

Early-stage Parkinson’s disease (PD) often features subtle sensorimotor integration deficits that may precede noticeable motor decline. This study examined how proprioceptive acuity, balance performance, and functional mobility relate to dual-task gait cost (DTC) in individuals with early-stage PD. Forty-six participants with idiopathic PD (Hoehn & Yahr stages I–II) completed assessments including passive ankle joint position sense testing for proprioception, the modified Clinical Test of Sensory Interaction on Balance (mCTSIB), and a reactive stepping task for balance. Gait was evaluated with the 10-Meter Walk Test under single- and dual-task (verbal fluency) conditions, and DTC was calculated from changes in gait speed. Greater proprioceptive error was significantly associated with slower dual-task gait speed (r = –0.52, p < 0.001), higher DTC (r = 0.47, p = 0.002), and increased stride time variability (r = 0.45, p = 0.003). In a multiple regression, proprioceptive error (β = 0.43, p < 0.001) and mCTSIB composite score (β = –0.40, p = 0.004) independently predicted DTC, with a mean of 17.3% (SD = 6.8). Participants who failed the reactive stepping task (n = 18) showed significantly greater proprioceptive error (mean = 4.7° vs. 2.9°, p < 0.001), slower dual-task gait speed (0.84 m/s vs. 1.06 m/s, p < 0.001), and lower verbal fluency scores (mean = 8.2 vs. 11.5 words, p < 0.001). These findings indicate that clinically measurable deficits in proprioception and balance are linked to impaired gait performance and reduced adaptability under cognitive load in early PD. Integrating sensorimotor assessments into early clinical evaluations may support timely, targeted interventions to mitigate mobility decline.

## Introduction

Parkinson’s disease (PD) is a progressive neurodegenerative disorder characterized primarily by motor symptoms such as bradykinesia, rigidity, tremor, and postural instability [1]. However, growing evidence suggests that non-motor impairments, particularly those affecting sensory processing and sensorimotor integration, are also critical contributors to functional decline in PD [2]. Mobility impairments, including gait disturbances and balance deficits, are common even in the early stages of the disease and are closely linked to reduced quality of life, increased fall risk, and loss of independence [3]. Dual-task gait interference—a reduced ability to walk while performing a concurrent cognitive task—is particularly pronounced in PD and serves as a marker of declining motor-cognitive integration [4]. Despite the clinical relevance of these impairments, assessments and interventions often focus predominantly on overt motor symptoms, overlooking the potential impact of underlying sensory deficits [5].

Proprioception, the sense of joint position and movement, plays a fundamental role in regulating gait and maintaining postural control [3]. Prior research has shown that individuals with PD exhibit reduced proprioceptive acuity, particularly in the lower limbs, which may impair motor planning and contribute to instability during walking and balance tasks [3,6,7]. These deficits appear to persist even in early disease stages, preceding observable motor decline [3,6,7]. Similarly, impairments in sensory integration—specifically the ability to process and reweight visual, vestibular, and somatosensory inputs—have been shown to compromise balance control under altered sensory conditions [8]. Clinical assessments such as the modified Clinical Test of Sensory Interaction on Balance (mCTSIB) and reactive stepping tasks have been used to capture these balance deficits [9,10]. Studies have also identified that individuals with PD display reduced adaptability in complex walking tasks, particularly during dual-task gait, where attentional demands may interfere with motor execution [11,12]. However, while previous studies have independently linked proprioceptive deficits or balance impairments with motor outcomes in PD, few have examined their combined or independent contributions to functional mobility under cognitive load [3,6,7]. Specifically, no previous studies have concurrently examined the independent and combined contributions of proprioceptive accuracy and clinically feasible balance measures, such as the mCTSIB, in predicting dual-task gait cost in early-stage PD. This represents a critical limitation in the literature, as understanding their relative and joint effects is essential for identifying clinically accessible markers of motor-cognitive interference.

A critical gap in the current literature is the limited understanding of how proprioception and sensory integration contribute to dual-task gait performance in early-stage PD. Most existing work has relied on either complex laboratory-based instrumentation or broad motor scales, limiting translation to routine clinical practice [13]. Moreover, while reactive balance responses have been shown to predict fall risk [14], their relationship to sensory and cognitive markers of gait dysfunction remains under-characterized [3]. There is a clear need to evaluate whether clinically feasible assessments of proprioceptive error and balance performance can identify individuals at risk for functional decline—particularly those who may appear relatively intact on standard motor evaluations. Addressing this knowledge gap is essential for refining early intervention strategies that target sensorimotor control mechanisms before significant disability occurs. Focusing specifically on individuals with early-stage PD (Hoehn and Yahr stages I–II) enabled the study to isolate sensorimotor integration deficits that emerge before pronounced motor complications and functional decline. This approach facilitated the identification of subtle impairments that may be masked in later stages by more severe motor symptoms and treatment-related effects, thereby supporting earlier and more targeted clinical intervention strategies.

The purpose of this study was to investigate the relationship between proprioceptive acuity, balance performance, and functional mobility in individuals with early-stage Parkinson’s disease using clinically accessible assessments. Specifically, the study aimed to (1) determine the association between proprioceptive error and gait performance, including single-task and dual-task conditions, and (2) evaluate whether balance performance and proprioception independently predict dual-task gait cost. It was hypothesized that greater proprioceptive error and impaired balance would be significantly associated with reduced gait performance and that both variables would independently predict higher dual-task cost, indicating reduced adaptability during cognitively demanding mobility tasks.

## Methods

### Study design, ethics, and settings

This cross-sectional study was conducted between 12^th^ January 2025 and 10^th^ December 2025 at the Movement Disorders Clinic, Department of Rehabilitation Sciences, King Khalid University, Aseer, Kingdom of Saudi Arabia. Ethical approval was obtained from the King Khalid University Institutional Review Board (KKU-166-2025-31) on 02^nd^ January 2025, and all participants provided written informed consent before enrollment. The study adhered fully to the ethical principles outlined in the Declaration of Helsinki. To minimize inter-examiner variability, all clinical and functional assessments were conducted by the same trained physiotherapist, following standardized protocols across all participants.

### Participants

Participants were recruited through consecutive sampling from the Movement Disorders Clinic at King Khalid University, Aseer, Kingdom of Saudi Arabia. Potential participants were identified during routine outpatient consultations or referred by neurologists affiliated with the clinic. A licensed physiotherapist specializing in neurological rehabilitation conducted the eligibility screening. A total of 62 individuals were initially screened for eligibility. Of these, 16 were excluded due to not meeting inclusion criteria or declining participation (n = 9 did not meet cognitive or mobility requirements; n = 7 declined to participate). The remaining 46 participants satisfied all eligibility criteria and were included in the final analysis. All participants met the diagnostic criteria for idiopathic Parkinson’s disease according to the United Kingdom Parkinson’s Disease Society Brain Bank Clinical Diagnostic Criteria [15], and were confirmed by a consultant neurologist. To ensure disease stage consistency, only individuals classified as Hoehn and Yahr stages I or II were included [15,16].

Inclusion criteria required that participants be aged 50–80 years, be ambulatory without the need for assistive devices, and have been on stable dopaminergic medication for at least 4 weeks before testing. Participants were required to have sufficient cognitive function to follow test instructions, operationalized as a score of ≥22 on the Montreal Cognitive Assessment (MoCA). Exclusion criteria included any history of neurological disorders other than PD (e.g., stroke, multiple sclerosis), musculoskeletal conditions significantly affecting gait or balance, uncorrected visual or vestibular deficits, and severe dyskinesia or motor fluctuations that would interfere with task performance. All participants underwent a standardized baseline assessment, including a clinical evaluation of motor severity using the Unified Parkinson’s Disease Rating Scale Part III (UPDRS-III) and cognitive screening using the MoCA, before completing the gait and balance testing protocols.

### Dual-task cost (DTC)

The primary outcome variable in this study was dual-task cost (DTC) [17], which quantifies the reduction in gait performance when a walking task is performed simultaneously with a cognitive task, compared to walking alone. This variable is widely recognized as a sensitive indicator of cognitive-motor interference and reduced functional adaptability in individuals with PD. In this study, DTC was calculated from gait speed measured under single- and dual-task conditions using the 10-Meter Walk Test (10MWT) [18], a validated and reliable clinical tool frequently employed in neurological rehabilitation to assess functional walking capacity. Dual-task cost was selected as the primary outcome rather than dual-task gait speed alone because it quantifies the relative change in performance between single- and dual-task conditions, thereby providing a normalized measure of cognitive-motor interference that accounts for individual differences in baseline gait ability.

Participants first completed the 10MWT in a single-task condition, walking at their usual comfortable pace over a marked 10-meter distance. To ensure accuracy, a 2-meter acceleration zone before the start line and a 2-meter deceleration zone after the finish line were included. Timing was conducted only over the central 10 meters, using a digital stopwatch accurate to 0.01 seconds. The test was then repeated under a dual-task condition, during which participants walked the same distance while performing a concurrent verbal fluency task, in which they named as many words as possible beginning with a designated letter (e.g., “F”) for the duration of the walk. Verbal fluency tasks are commonly used in PD dual-task research because they impose executive and attentional demands relevant to real-life scenarios.

Each condition was performed twice, and the average gait speed in meters per second (m/s) was calculated by dividing the distance (10 meters) by the recorded time in seconds. Dual-task cost was then computed using the following formula: DTC (%) = [(Single-task gait speed – Dual-task gait speed)/ Single-task gait speed] × 100. This value represents the percentage decrease in gait speed due to cognitive load, with higher percentages indicating greater interference and reduced gait automaticity. To ensure standardization and minimize confounding factors, all gait assessments were performed during the participants’ on-medication phase (60–90 minutes after dopaminergic intake), in a quiet, well-lit corridor free from environmental distractions. All participants were given clear, standardized instructions and a practice trial before data collection to ensure comprehension and familiarity with the task. This approach ensured the DTC metric reflected genuine motor-cognitive interaction rather than variability due to unfamiliarity or testing conditions.

### Proprioceptive accuracy: Passive joint position sense testing

Proprioceptive error was measured as a key independent variable using a standardized passive joint position sense (JPS) test at the ankle [19], focusing on participants’ accuracy in perceiving limb position without visual input. This method is widely used in neurological populations, including individuals with PD, and has been shown to be both clinically feasible and sensitive to subtle sensory deficits [20,21].

Participants were seated comfortably in a standardized position with hips and knees flexed at approximately 90 degrees. Both lower limbs were supported, and care was taken to minimize extraneous movement. The test was conducted with participants blindfolded and wearing noise-canceling headphones to eliminate visual and auditory cues, thereby isolating proprioceptive input. The examiner passively moved the non-tested (reference) ankle to a target angle within the participant’s normal range of dorsiflexion or plantarflexion (typically 10–20°). After maintaining the target position for 5 seconds to allow sensory encoding, the limb was returned to neutral. The participant was then instructed to replicate the same position with the tested (active) limb as accurately as possible, and the limb was also moved passively by the examiner to reduce motor involvement. The participant signaled when they perceived the limb to be in the same position.

The absolute angular difference between the replicated and target positions was measured using a standard manual goniometer aligned with anatomical landmarks (the lateral malleolus, fibular head, and fifth metatarsal) to ensure consistency. This process was repeated for three trials per limb, alternating sides, and the mean absolute error (in degrees) from the three trials was calculated and used for analysis. Proprioceptive error values obtained from both limbs were averaged to yield a single representative measure per participant for statistical analysis. No feedback was provided during or between trials to avoid motor learning effects. The testing order was counterbalanced across participants to control for fatigue or order effects. The lower limb was chosen based on its relevance to postural control and gait regulation. Testing was conducted at a consistent time of day during the participants’ “on” medication state to reduce variability due to motor fluctuations.

### Balance and sensory integration performance

Balance performance was assessed using the MCTSIB [9]. This standardized clinical tool evaluates postural stability under four progressively challenging sensory conditions: eyes open on a firm surface; eyes closed on a firm surface; eyes open on a compliant foam surface; and eyes closed on a compliant foam surface [9]. Each trial lasted a maximum of 30 seconds, and participants were instructed to stand with feet together and arms at their sides, maintaining balance without stepping, opening their eyes (in closed-eye conditions), or requiring support. A composite score out of 120 seconds was calculated by summing the durations successfully maintained across all four conditions, with higher scores indicating more effective sensory integration and postural control. Standardized instructions were given before testing, and a single trained examiner conducted all assessments in a quiet, well-lit clinic space using a medium-density foam pad for the foam-surface trials. In addition to static balance, reactive balance was evaluated using a perturbation-stepping response test adapted from Mancini et al. (2012), in which participants stood upright and were subjected to unexpected manual perturbations applied to the shoulders in random directions. The intensity of perturbations was standardized by applying consistent manual force sufficient to elicit a stepping response while ensuring participant safety, as determined through prior practice trials and adherence to a predefined protocol used uniformly across all participants. A failed response was recorded if participants required external assistance, took more than one step, or exhibited a delayed stepping response of more than 1 second. Each participant completed three perturbation trials, and responses were monitored closely to assess compensatory stepping ability.

### Motor symptom severity

Motor symptom severity was assessed using the Unified Parkinson’s Disease Rating Scale, Part III (UPDRS-III) [22], which is the motor subsection of the Movement Disorder Society’s comprehensive PD assessment tool. This clinician-administered scale includes 18 items that assess key motor symptoms of PD, including resting tremor, action tremor, rigidity, finger-tapping, hand movements, rapid alternating movements of the hands, lower-limb agility, rising from a chair, posture, gait, postural stability, and bradykinesia. Each item is rated on a scale from 0 (normal) to 4 (severe), with total scores ranging from 0 to 108; higher scores indicate greater motor impairment. All assessments were performed by trained physiotherapists or clinicians certified in the standardized administration of the UPDRS, following Movement Disorder Society (MDS) guidelines to ensure consistency, inter-rater reliability, and clinical validity [23].

### Cognitive function

Cognitive function was screened using the Montreal Cognitive Assessment (MoCA) [24], a widely validated cognitive screening tool sensitive to mild cognitive impairment in PD. The MoCA evaluates domains including executive function, visuospatial skills, attention, memory, language, and orientation. Scores range from 0 to 30, with a cut-off of 26 commonly used to indicate normal cognition in the general population; however, for inclusion in this study, a score of ≥22 was required to ensure participants retained sufficient cognitive capacity to understand and follow task instructions while also allowing the inclusion of individuals with mild cognitive deficits. The MoCA was administered in a quiet environment by trained examiners following the standardized scoring protocol [25].

### Demographic and clinical covariates

Additional demographic and clinical covariates included age (years), sex (male/female), disease duration (years since confirmed clinical diagnosis), and levodopa equivalent daily dose (LEDD). LEDD was calculated using the standardized conversion formulas for dopaminergic medications, as outlined by Tomlinson et al. (2010), to quantify total antiparkinsonian medication burden in milligrams. These variables were obtained through structured participant interviews and, where available, confirmed through medical records. Each was selected for its potential to influence motor and gait-related performance and was included in the analysis to adjust for confounding effects on the outcome measures. Collectively, these measures provided a comprehensive clinical profile necessary to contextualize sensorimotor and functional mobility performance in early-stage PD.

### Sample size calculation

A priori sample size estimation was conducted using G*Power 3.1.9.7. For the primary analysis assessing the correlation between proprioceptive error and dual-task gait speed, a two-tailed for the primary analysis assessing the correlation between proprioceptive error and dual-task gait speed, a two-tailed Pearson correlation was assumed with a medium effect size (r = 0.40), an alpha level of 0.05, and statistical power of 0.80. This yielded a required sample size of 46 participants, which was achieved.

### Data analysis

All statistical analyses were conducted using IBM SPSS Statistics for Windows, version 24.0 (IBM Corp., Armonk, NY, USA). Data were first screened for normality using the Shapiro–Wilk test and visual inspection of histograms and Q-Q plots, confirming that all continuous variables were normally distributed; therefore, parametric statistical methods were applied. Descriptive statistics were used to summarize participant characteristics and study variables, with continuous variables presented as means and standard deviations, and categorical variables as frequencies and percentages. Independent-samples t-tests were used to compare continuous variables between participants who passed and failed the reactive stepping task, and chi-square tests were used for categorical group comparisons. Pearson correlation coefficients were calculated to examine associations between proprioceptive error and gait-related variables, including dual-task gait speed, dual-task cost, step length, cadence, stride-time variability, and double-support time. To evaluate the extent to which proprioceptive and balance impairments predicted functional mobility outcomes, a multiple linear regression analysis was conducted with dual-task cost as the dependent variable and proprioceptive error, mCTSIB composite score, age, and disease duration entered as independent variables. Multicollinearity was assessed using variance inflation factors to ensure model validity. All statistical tests were two-tailed, and p-values < 0.05 were considered statistically significant.

## Results

Participants demonstrated characteristics consistent with early-stage PD, with a mean age of 66.42 years and a disease duration of approximately 3.5 years (Table 1). The sample was balanced across Hoehn & Yahr stages I and II, and most participants exhibited relatively preserved global cognition, as reflected in a mean MoCA score of 25.38. Motor symptom severity was mild to moderate, with a mean UPDRS-III score of 21.65, and the average daily levodopa equivalent dose was 312.45 mg. Functional mobility assessments indicated a reduction in gait speed during dual-task walking compared to single-task conditions (1.00 vs. 1.23 m/s), corresponding to a clinically relevant dual-task cost of 18.70%. Sensorimotor metrics showed a mean proprioceptive error of 4.21°, and balance performance, as measured by the mCTSIB, averaged 25.48 seconds. Notably, over one-third of participants (34.78%) failed the reactive stepping test, suggesting early balance control impairments despite relatively mild disease severity.

**Table 1:** Demographic and Clinical Characteristics of Participants.

Variable	Mean ± SD or n (%)	95% CI
Age (years)	66.42 ± 7.81	64.38–68.46
Sex (Male/Female)	28 (60.87%)/ 18 (39.13%)	–
Disease duration (years)	3.46 ± 1.92	2.91–4.01
Hoehn & Yahr stage I/II	24 (52.17%)/ 22 (47.83%)	–
MoCA score	25.38 ± 2.71	24.58–26.18
UPDRS-III (motor section)	21.65 ± 7.94	19.48–23.82
Daily Levodopa Equivalent Dose (mg)	312.45 ± 145.26	271.14–353.76
10-Meter Walk Test (single-task, m/s)	1.23 ± 0.26	1.15–1.31
10-Meter Walk Test (dual-task, m/s)	1.00 ± 0.21	0.93–1.07
Dual-task cost (%)	18.70 ± 5.54	17.13–20.27
Proprioceptive error (°)	4.21 ± 1.16	3.92–4.50
mCTSIB composite score (s)	25.48 ± 6.39	23.82–27.14
Reactive stepping failure (n, %)	16 (34.78%)	–

MoCA; Montreal Cognitive Assessment; UPDRS-III; Unified Parkinson’s Disease Rating Scale Part III; mCTSIB; Modified Clinical Test of Sensory Interaction on Balance; SD; Standard Deviation; CI; Confidence Interval; DTC; Dual-task cost.

**Fig 1:**
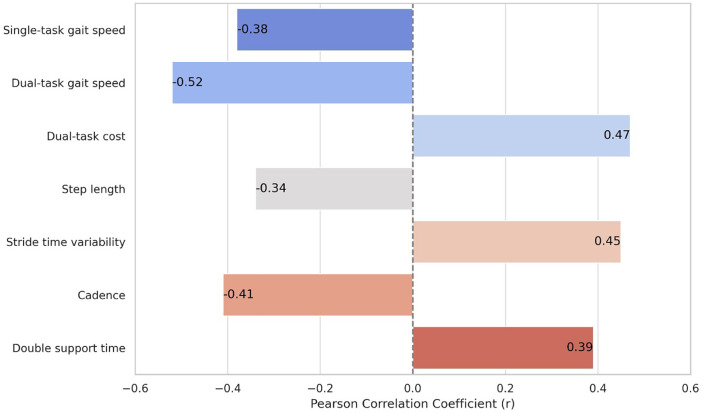
Correlation coefficients between proprioceptive error and key gait parameters in early-stage Parkinson’s disease.

Proprioceptive error was significantly associated with multiple gait parameters, indicating a clear link between sensory dysfunction and impaired functional mobility in early-stage PD (Table 2 and Fig 1). Greater proprioceptive error correlated with slower gait speed during both single-task (r = –0.38, *p* = 0.006) and dual-task conditions (r = –0.52, *p* < 0.001), and with a higher dual-task cost (r = 0.47, *p* = 0.002), suggesting diminished adaptability under cognitive load. Additional correlations revealed that increased proprioceptive error was associated with shorter step length (r = –0.34, *p* = 0.014), higher stride time variability (r = 0.45, *p* = 0.003), reduced cadence (r = –0.41, *p* = 0.004), and prolonged double support time (r = 0.39, *p* = 0.005).

**Table 2:** Correlations Between Proprioceptive Error and Gait Parameters.

Variable	Mean ± SD	Pearson r	95% CI	p-value
Single-task gait speed (m/s)	1.23 ± 0.26	−0.38	−0.60 to −0.12	0.006
Dual-task gait speed (m/s)	1.00 ± 0.21	−0.52	−0.70 to −0.29	<0.001
Dual-task cost (%)	18.70 ± 5.54	0.47	0.21 to 0.65	0.002
Step length (cm)	58.34 ± 6.29	−0.34	−0.57 to −0.07	0.014
Stride time variability (%)	3.12 ± 0.74	0.45	0.19 to 0.64	0.003
Cadence (steps/min)	108.42 ± 10.65	−0.41	−0.62 to −0.15	0.004
Double support time (%)	32.58 ± 4.61	0.39	0.13 to 0.61	0.005

SD; Standard Deviation; CI; Confidence Interval; DTC; Dual-task cost; m/s; meters per second; cm; centimeters.

**Fig 2:**
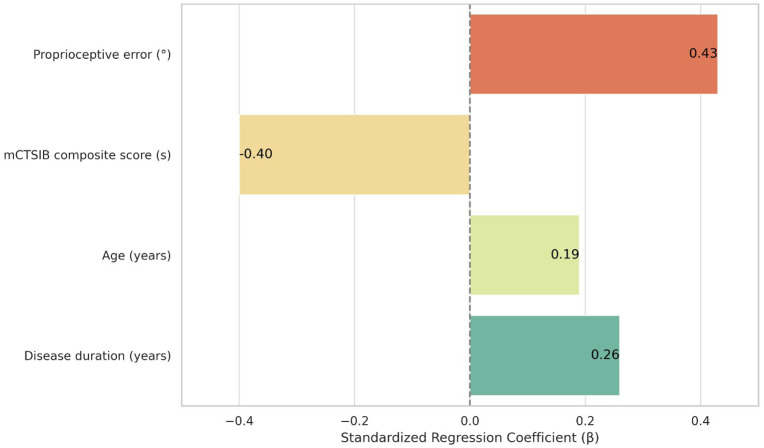
Standardized regression coefficients (β) predicting dual-task cost from proprioceptive error, balance performance, age, and disease duration in individuals with early-stage Parkinson’s disease.

Proprioceptive error and balance performance were significant predictors of dual-task cost, indicating their independent contributions to functional mobility impairments in early PD (Table 3 and Fig 2). Higher proprioceptive error was strongly associated with increased dual-task cost (β = 0.43, *p* < 0.001), while lower mCTSIB composite scores were also significantly associated with greater cost (β = –0.40, *p* = 0.004), highlighting the role of sensory integration in gait performance under cognitive load. Disease duration showed a modest but statistically significant effect (β = 0.26, *p* = 0.034), whereas age was not a significant predictor (β = 0.19, *p* = 0.183).

**Table 3:** Linear Regression Predicting Dual-Task Cost from Balance and Proprioception.

Predictor Variable	Unstandardized Coefficient (B)	Standard Error	Standardized Coefficient (β)	95% CI for B	p-value
Proprioceptive error (°)	2.35	0.71	0.43	0.94 to 3.76	<0.001
mCTSIB composite score (s)	−0.41	0.14	−0.40	−0.69 to −0.13	0.004
Age (years)	0.12	0.09	0.19	−0.06 to 0.30	0.183
Disease duration (years)	0.28	0.13	0.26	0.02 to 0.54	0.034

mCTSIB; Modified Clinical Test of Sensory Interaction on Balance; SD; Standard Deviation; CI; Confidence Interval; B; Unstandardized Coefficient; β; Standardized Coefficient.

**Fig 3:**
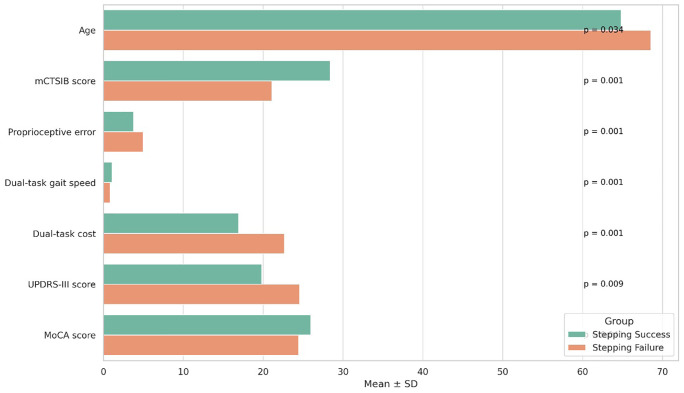
Comparison of sensorimotor, balance, and functional mobility outcomes between participants with successful versus failed reactive stepping responses in early-stage Parkinson’s disease.

Participants who failed the reactive stepping task demonstrated significantly greater impairments across multiple domains of sensorimotor and functional performance compared to those who succeeded (Table 4 and Fig 3). Stepping failure was associated with older age (*p* = 0.034), lower mCTSIB composite scores (*p* < 0.001), and greater proprioceptive error (*p* < 0.001), indicating reduced sensory integration and balance control. Functional mobility was also compromised in the failure group, with slower dual-task gait speed and higher dual-task cost (both *p* < 0.001), suggesting diminished adaptability under cognitive load. Additionally, higher UPDRS-III scores (*p* = 0.009) and lower MoCA scores (*p* = 0.041) were observed, reflecting more severe motor and cognitive symptoms.

**Table 4:** Group Comparison: Reactive Stepping Failure vs. Success.

Variable	Stepping Success (n = 30) Mean ± SD	Stepping Failure (n = 16) Mean ± SD	95% CI of Difference	p-value
Age (years)	64.83 ± 7.25	68.56 ± 8.01	−6.89 to −0.36	0.034
Disease duration (years)	3.15 ± 1.74	4.03 ± 2.15	−1.71 to 0.01	0.052
mCTSIB composite score (s)	28.41 ± 5.93	21.11 ± 4.87	4.23 to 10.31	<0.001
Proprioceptive error (°)	3.78 ± 0.99	4.96 ± 1.01	−1.65 to −0.64	<0.001
Dual-task gait speed (m/s)	1.08 ± 0.20	0.84 ± 0.17	0.13 to 0.33	<0.001
Dual-task cost (%)	16.92 ± 4.84	22.64 ± 5.22	−8.59 to −2.15	<0.001
UPDRS-III score	19.83 ± 6.54	24.56 ± 8.32	−9.00 to −1.12	0.009
MoCA score	25.97 ± 2.46	24.44 ± 2.97	0.12 to 3.04	0.041

mCTSIB; Modified Clinical Test of Sensory Interaction on Balance; MoCA; Montreal Cognitive Assessment; UPDRS-III; Unified Parkinson’s Disease Rating Scale Part III; SD; Standard Deviation; CI; Confidence Interval; DTC; Dual-task cost; m/s; meters per second.

## Discussion

This study aimed to examine the relationship between proprioceptive acuity, balance performance, and functional mobility in individuals with early-stage PD using practical assessments. Specifically, the objectives were to determine whether proprioceptive deficits and impaired balance were associated with altered gait performance, particularly under dual-task conditions, and to explore whether these sensorimotor impairments predicted functional limitations. The findings showed significant links between higher proprioceptive error and various indicators of gait dysfunction, including slower gait speed, increased dual-task cost, and signs of gait instability. Balance impairment, measured by sensory integration ability and reactive stepping capacity, also proved to be a key factor in mobility limitations, particularly when cognitive load was present. Regression analysis further confirmed that proprioceptive and balance measures independently predicted dual-task cost, highlighting their influence on gait adaptability. These findings should be interpreted as indicative of associations rather than predictive relationships, as the cross-sectional design does not permit conclusions regarding temporal or causal links between sensorimotor impairments and mobility decline. Additionally, participants who failed the reactive stepping task showed greater deficits across sensorimotor, motor, and cognitive domains, underscoring the functional importance of compensatory balance mechanisms in early PD. These results underscore the importance of focusing on sensorimotor integration in early intervention strategies to help maintain mobility and reduce fall risk.

The significant association between proprioceptive error and gait performance observed in this study aligns with existing evidence that somatosensory dysfunction impairs mobility in PD [19]. Previous research has shown that individuals with early-stage PD often exhibit reduced proprioceptive processing, which hampers the ability to generate accurate motor responses during walking [26; 27]. Impaired lower-limb proprioception has been explicitly associated with slower gait speed and greater gait variability, likely due to disrupted feedback control required for step regulation [3,28]. Additionally, dual-task conditions tend to worsen gait impairments in PD, especially when sensory deficits are present, as attention is diverted from sensorimotor monitoring [29,30]. The current findings support this relationship, as increased proprioceptive error was linked to higher dual-task cost, suggesting reduced gait adaptability under cognitive load [11,31]. This pattern aligns with earlier studies indicating that proprioceptive dysfunction may serve as an early sign of functional decline, contributing to limited mobility even in the absence of significant motor deterioration [3]. Cognitive function may also have contributed to variability in dual-task performance, as reflected in the lower MoCA scores observed in participants with poorer functional outcomes [26]. Even mild cognitive impairment, particularly in executive function and attention, can reduce the ability to allocate cognitive resources effectively during simultaneous motor and cognitive tasks [27]. This limitation may exacerbate dual-task interference, leading to greater reductions in gait speed and increased dual-task cost. These findings highlight the importance of considering cognitive status when interpreting dual-task gait performance in individuals with early-stage PD.

The observed connection between impaired balance and increased dual-task cost further emphasizes the importance of sensory integration in gait control. The significant impact of mCTSIB scores on dual-task cost supports earlier research indicating that postural control under altered sensory conditions is a sensitive marker of sensorimotor integration problems in PD [32,33]. Difficulty maintaining balance when sensory input is limited suggests a reduced ability to reweight or integrate visual, vestibular, and somatosensory cues—an issue known to impair motor performance during complex tasks [32]. The additional cognitive load of dual-task walking may therefore overwhelm an already weakened balance system, leading to disproportionate drops in gait speed and efficiency [34]. Moreover, the group comparisons showed that individuals who failed the reactive stepping test not only had poorer balance and proprioception but also exhibited more significant motor and cognitive deficits [35]. This supports earlier findings by Monaghan et al. [35], who noted that reactive balance responses are highly predictive of fall risk and are associated with both sensorimotor and executive functions in early PD. Overall, these findings highlight the clinical value of incorporating sensory-focused assessments into routine gait and balance evaluations to better detect early functional changes in PD [36].

### Clinical significance

The findings of this study provide clinically actionable insights by demonstrating that proprioceptive error and impaired balance performance are independently associated with reduced gait efficiency and increased dual-task cost in individuals with early-stage PD. Importantly, these associations were identified using low-cost, clinically feasible tools, such as joint position sense testing and the mCTSIB, making the results directly translatable to routine outpatient neurological physical therapy practice. The identification of sensorimotor integration deficits in early PD, particularly under cognitively demanding gait conditions, supports prioritizing early, targeted interventions focused on proprioceptive training and sensory-based balance strategies. Furthermore, the strong relationship between reactive stepping failure and functional impairment underscores the utility of incorporating dynamic balance testing to identify individuals at elevated risk of mobility decline. It falls, even in the early stages of the disease. However, these observations reflect cross-sectional associations and should not be interpreted as evidence of prospective risk prediction without longitudinal validation.

### Limitations of the study and areas of future research

This study was limited by its cross-sectional design, which precluded causal inference regarding the relationship between sensorimotor impairments and functional mobility outcomes. Additionally, the sample was limited to individuals with early-stage PD and relatively preserved cognitive function, which may limit generalizability to more advanced stages or cognitively impaired populations. The use of clinical rather than instrumented measures, while practical, may have reduced the precision with which subtle gait and balance abnormalities are captured. Future research should include longitudinal studies to determine the predictive validity of proprioceptive and balance deficits on fall risk and disease progression. Incorporating objective sensor-based technologies and expanding the sample to include diverse PD subtypes and stages would further enhance the generalizability and mechanistic understanding of sensorimotor contributions to mobility decline.

## Conclusions

This study demonstrated that greater proprioceptive error and impaired balance performance are significantly associated with functional mobility limitations, particularly under dual-task conditions, in individuals with early-stage PD. Both proprioceptive deficits and reduced sensory integration capacity were independently predictive of dual-task cost, and individuals with reactive stepping failure exhibited more pronounced impairments across motor, sensory, and cognitive domains. These findings underscore the relevance of sensorimotor integration assessments in early-stage PD and support the clinical value of implementing accessible, non-instrumented measures to inform early intervention strategies to preserve gait function and balance.
